# Two-Dimensional Cell Separation: a High-Throughput Approach to Enhance the Culturability of Bacterial Cells from Environmental Samples

**DOI:** 10.1128/spectrum.00007-22

**Published:** 2022-04-25

**Authors:** Krishna K. Yadav, Yogesh Nimonkar, Bhagyashri J. Poddar, Lochana Kovale, Isita Sagar, Yogesh Shouche, Hemant J. Purohit, Anshuman A. Khardenavis, Stefan J. Green, Om Prakash

**Affiliations:** a National Centre for Microbial Resource, National Centre for Cell Science, Pune, Maharashtra, India; b Environmental Biotechnology and Genomics Division, CSIR-National Environmental Engineering Research Institute, Nagpur, Maharashtra, India; c Academy of Scientific and Innovative Research (AcSIR), Ghaziabad, Uttar Pradesh, India; d Genomics and Microbiome Core Facility, Rush University Medical Center, Chicago, Illinois, USA; University of Massachusetts Amherst

**Keywords:** not yet cultured, culturomics, cultivability, bacteria, environmental samples

## Abstract

Culture-independent sequence data from various environmental samples have revealed an immense microbial diversity of environmental, clinical, and industrial importance that has not yet been cultured. Cultivation is imperative to validate findings emerging from cultivation-independent molecular data and exploit the isolated organisms for biotechnological purposes. Efforts have been made to boost the cultivability of microbes from environmental samples by use of a range of techniques and instrumentation. The manuscript presents a novel yet simple and innovative approach to improving the cultivability of natural microorganisms without sophisticated instrumentation. By employing gradient centrifugation combined with serial dilution (“two-dimensional cell separation”), significantly higher numbers of genera (>2-fold higher) and species (>3-fold higher) were isolated from environmental samples, including soil, anaerobic sludge, and landfill leachate, than from using serial dilution alone. This simple and robust protocol can be modified for any environment and culture medium and provides access to untapped microbial diversity.

**IMPORTANCE** In the manuscript, we have developed a novel yet simple and innovative approach to improving the cultivability of natural microorganisms without sophisticated instrumentation. The method used gradient centrifugation combined with serial dilution (two-dimensional cell separation) to improve taxum recovery from samples. This simple and robust protocol can be modified for any environment and culture medium and provides access to untapped microbial diversity. This approach can be incorporated with less labor and complexity in laboratories with minimal instrumentation. As cultivation is a workflow that is well suited to lower-resource microbiology labs, we believe improvements in cultivability can increase opportunities for scientific collaborations between low-resource labs and groups focused on high-resource cultivation-independent methodologies.

## INTRODUCTION

Most modern microbial ecology studies utilize cultivation-independent techniques, including PCR, quantitative PCR (qPCR), 16S rRNA gene amplicon sequencing, shotgun metagenome sequencing, functional metagenomics, and metatranscriptome sequencing (1). Although somewhat neglected, the cultivation of microorganisms is a critical companion to cultivation-independent molecular analyses. In addition to their use as biotechnological agents, cultivated microorganisms can be used to verify hypotheses about environmental microorganisms' physiological capabilities and define metabolic activity under a broad range of environmental conditions (2–5). For example, a novel organism from the genus *Nitrospira*, capable of complete oxidation of ammonia to nitrate, was recently isolated (6). Historically, *Nitrospira* has been considered only capable of nitrite oxidation, and the authors consider that the true role of bacteria from the genus *Nitrospira* has been underestimated. Similarly, coupled cultivation and genome sequencing of bacteria from the genus *Rhodanobacter* demonstrated that not all members of the genus were capable of denitrification from nitrate (7). *Rhodanobacter* isolates testing negative for growth with nitrate as the sole electron acceptor were found to have complete denitrification pathways, with the exception of nitrate reductase genes, suggesting denitrification potential for all members of the genus. Microbial isolates can also be used to generate complete genomes that can serve as references for additional cultivation-independent analyses (8). The cultivation and isolation of environmental microorganisms, however, is highly challenging, and most environmental microorganisms have yet to be cultivated (9–13).

Furthermore, the most relevant microbial taxa, i.e., those that are abundant *in situ* or that catalyze critical metabolic processes, are not easily cultivated, while less relevant but culture-amendable taxa are recovered (14). The large uncultivated fraction of microbial communities has been observed microscopically (10), leading to the great plate count anomaly (15–18) that has also been more recently validated through metagenomic sequencing. Here, too, cultivation and metagenome sequencing are best used in tandem: metagenome sequencing approaches are not well suited to the characterization of low abundance taxa, regardless of the role they play in a given ecosystem (12, 19). Thus, cultivation of the not yet cultured is essential for an improved understanding of microbial communities.

To overcome difficulties in cultivation, environmental microbiologists have employed a broad range of sophisticated techniques. Beyond modification of cultivation media and growth conditions (e.g., reduced organic content in media has been shown to improve the isolation of relevant microbial taxa) (20), performing cell separation before cultivation has proved to be among the most successful cultivation techniques. In the most basic form of cell separation, dilution to extinction is performed, plating the dilutions to recover greater colony diversity (21, 22). Similarly, selective filtration has been performed; for example, syringe filters ranging from 5 μm pore size to 0.2 μm have been used successively, and each microbial-size fraction was used to inoculate media (19, 23). Further fractionation of cells has been performed by encapsulation of single cells in permeable materials to allow for growth in the absence of other microorganisms. Such approaches often utilize sophisticated materials and instrumentation, including the I-chip (24) and gel microdroplets (25) and *in situ* incubation after encapsulation or separation. Still, many more techniques are being developed in culturomics for obtaining an extensive insight into natural diversity by maximizing cultivability (25–34). However, with the advancement in cultivation techniques, there is often a decrease in simplicity, leading to reduced method uptake across the field and inaccessibility to laboratories with fewer resources. In the current study, we have developed a simple cultivation method that can significantly expand the accessible microbial fraction and be performed without specialized instrumentation.

## RESULTS

**FIG 1:**
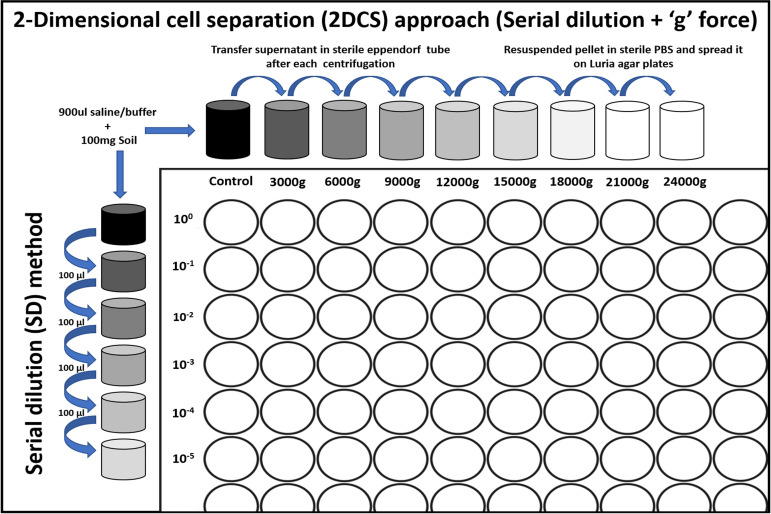
Graphical representation the 2-dimensional cell separation (2DCS) method. A cell suspension prepared from the source sample was separated using gradient centrifugation at centrifugal forces (*g*) increasing from left to right. Pellets obtained after each centrifugation were resuspended, serially diluted, and plated. Supernatants were subjected to higher-level centrifugal forces, and the process was repeated. Samples in this study were subject to centrifugal force increments of 3,000 × *g* up to 21,000 × *g*; serial order-of-magnitude dilutions were performed to 10^−5^ for all cell pellets.

**FIG 2:**
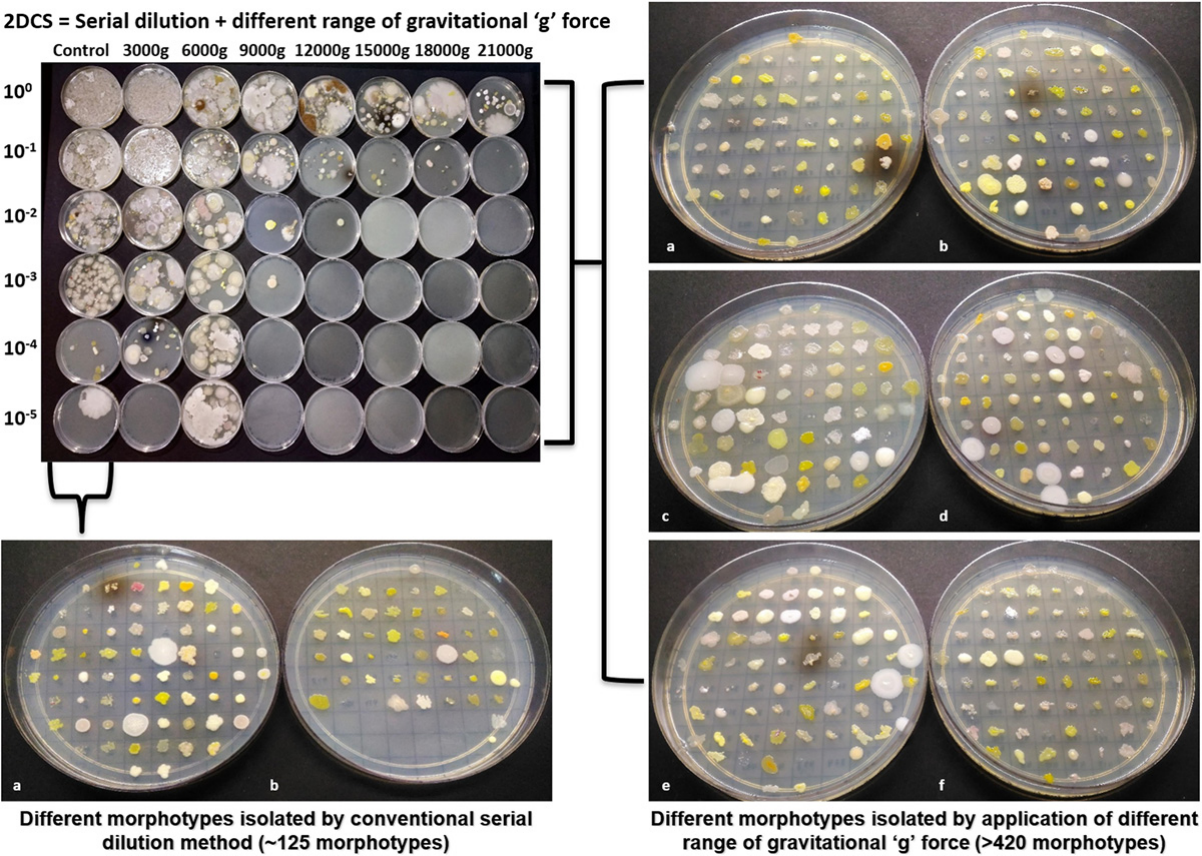
Images of a representative cultivation effort for a single sample using the SD and 2DCS methods (top left corner). A colony library for isolates obtained using the standard serial dilution (SD) methodology is shown at the bottom left. A colony library for the same sample but using the 2DCS method is shown on the right.

The novel cultivation approach designed and used in the current study is presented in Fig. 1 and 2. A comparative overview of the number of taxa obtained from different samples using serial dilution (SD) and the novel 2-dimensional cell separation (2DCS) approach is presented in Table 1. A comparison of cultivation techniques was performed on three different sample types, including soil (aerobic), digester sludge (anaerobic), and landfill leachate (aerobic). Across all three sample types, the 2DCS method captured a wider range of bacterial diversity relative to SD plating at the taxonomic levels of genus (*P* = 0.014), species (*P* = 0.003), and unique morphotypes (*P* = 0.005). Overall, agricultural soil had the highest level of cultivated bacterial diversity, while that of the anaerobic sludge was the lowest (Table 1). Relatively little overlap in isolated taxa was observed between sample types.

**TABLE 1:** Comparison of cultivated microbial diversity achieved with SD and 2DCS approaches for environmental samples^*a*^

Sample type or statistical value	Total no. of isolates found with:		Total no. of genera found with:		Total no. of species found with:		No. of genera found with each method within phylum:							
							*Proteobacteria*		*Bacteroidetes*		*Actinobacteria*		*Firmicutes*	
	SD	2DCS	SD	2DCS	SD	2DCS	SD	2DCS	SD	2DCS	SD	2DCS	SD	2DCS
Sample type														
Agricultural soil^*b*^	110	604	35	82	73	227	16	39	2	3	10	23	7	17
Anaerobic digester sludge^*c*^	70	380	17	53	37	162	8	24	2	5	3	10	4	15
Landfill leachate^*c*^	75	425	17	63	36	205	12	24	3	4	0	15	2	20
Statistical value														
Avg	85	469.67	23	66	48.67	198	12	29	2.33	4	4.33	16	4.33	17.33
SD	21.79	118.49	10.39	14.73	21.08	33.06	4	8.66	0.58	1	5.13	6.55	2.51	2.51
SE	12.58	68.41	6	8.50	12.17	19.08	2.31	5	0.33	0.58	2.96	3.78	1.45	1.45
F statistic	30.58		17.07		43.52		9.53		6.25		5.89		40.03	
*P* value	0.005		0.014		0.003		0.037		0.067		0.072		0.003	

a SD, serial dilution; 2DCS, two-dimensional cell separation, which is the increasing centrifugal force separation plus serial dilution.

b For the agricultural soil isolates, bacterial 16S rRNA gene amplicon sequences were acquired using DNA extraction of individual colonies, PCR amplification, and capillary electrophoresis sequencing.

c For digester and leachate samples, isolates were grown in liquid media and pooled, and a single DNA extraction was performed. Mixed genomic DNA was PCR amplified and sequenced using an Illumina MiSeq sequencer.

Cultivation by the 2DCS method yielded roughly 2- to 4-fold the number of genera recovered and roughly 3- to 6-fold the number of species recovered (Table 1). All isolates belonged to one of four phyla, including *Proteobacteria*, *Bacteroidetes*, *Actinobacteria*, and *Firmicutes*, and the number of genera detected in each phylum from the 2DCS isolation relative to the SD method was significantly higher or trending higher (*P* = 0.003 to 0.072) for all four phyla (Table 1). At the taxonomic level of genus, all genera isolated in the SD method were also recovered in the 2DCS method. Bacterial genera isolated from soil, sludge, and leachate are shown in Tables 2 to 4, respectively, and bacterial species isolated are shown in Tables S1 to S3 in the supplemental material, respectively. Using the 2DCS approach, bacterial genera that are rarely isolated using traditional cultivation methods, and with few repository representatives, were isolated. Among the samples tested, we identified isolates from 13 genera for which there are fewer than 5 validly described species (35). These genera included *Leclercia* (1 species), *Brachymonas* (3 species), *Krasilnikoviella* (2 species), *Morganella* (2 species), *Pseudoduganella* (3 species), *Methylocella* (3 species), *Paucisalibacillus* (2 species), and *Piscicoccus* (1 species) from soil; *Okibacterium* (2 species), *Renibacterium* (2 species), *Xylella* (2 species), and *Zimmermannella* (4 species) from landfill leachate; and *Kytococcus* (3 species) and *Xylella* (2 species) from anaerobic sludge. In addition, based on 16S rRNA gene sequence similarity as assessed by BLAST analysis, we identified numerous potentially novel isolates with <98.5% similarity to described species in GenBank (Table S5). Using the SD method, we identified 40 taxa with <98.5% similarity to described species and 240 such taxa using the 2DCS method from across all samples.

**TABLE 2:** Bacterial genera cultivated from soil using SD and 2DCS cultivation methods

Genera cultivated by the SD method			Genera cultivated by the 2DCS method		
*Agrobacterium*	*Priestia*	Acinetobacter	*Domibacillus*	*Methylocella*	*Pseudoduganella*
*Agromyces*	*Pseudochrobactrum*	*Actinoplanes*	*Ensifer*	*Microbacterium*	Pseudomonas
*Bacillus*	Pseudomonas	*Agrobacterium*	Enterobacter	*Micrococcus*	*Pseudoxanthomonas*
*Brachybacterium*	*Pseudoxanthomonas*	*Agromyces*	*Enterococcus*	*Morganella*	*Psychrobacillus*
Brucella	*Psychrobacillus*	*Alcaligenes*	*Erwinia*	*Neobacillus*	*Rhizobium*
*Cellulomonas*	*Rhizobium*	*Alkalihalobacillus*	*Glutamicibacter*	*Neorhizobium*	*Rhodobacter*
*Cellulosimicrobium*	*Rhodococcus*	*Ancylobacter*	*Gordonia*	*Niallia*	*Rhodococcus*
*Chryseobacterium*	*Shigella*	*Arthrobacter*	*Isoptericola*	*Nitrincola*	*Shigella*
*Erwinia*	*Sphingobacterium*	*Bacillus*	*Janibacter*	*Nocardia*	*Shinella*
*Glutamicibacter*	*Sphingomonas*	*Bosea*	*Kineosporia*	*Nocardioides*	*Sphingobacterium*
*Gordonia*	*Sphingopyxis*	*Brachybacterium*	Klebsiella	*Novosphingobium*	*Sphingobium*
*Isoptericola*	*Stenotrophomonas*	*Brachymonas*	*Knoellia*	*Ochrobactrum*	*Sphingomonas*
*Luteimonas*	*Streptomyces*	*Brevundimonas*	*Krasilnikoviella*	*Paenibacillus*	*Sphingopyxis*
*Lysinibacillus*	*Terribacillus*	Brucella	*Leclercia*	*Pantoea*	*Sporosarcina*
*Lysobacter*		*Cellulomonas*	*Leucobacter*	*Paracoccus*	*Stenotrophomonas*
*Metabacillus*		*Cellulosimicrobium*	*Luteimonas*	*Paucisalibacillus*	*Streptomyces*
*Methylocella*		*Chryseobacterium*	*Lysinibacillus*	*Pedobacter*	*Terribacillus*
*Microbacterium*		*Citrobacter*	*Lysobacter*	*Piscicoccus*	*Ureibacillus*
*Neobacillus*		*Curtobacterium*	*Massilia*	*Priestia*	*Xanthomonas*
*Neorhizobium*		*Cytobacillus*	*Mesobacillus*	*Pseudarthrobacter*	
*Novosphingobium*		*Devosia*	*Metabacillus*	*Pseudochrobactrum*	
Total=35		Total=82			

**TABLE 3:** Bacterial genera cultivated from sludge using the SD and 2DCS cultivation methods

Genera cultivated by the SD method	Genera cultivated by the 2DCS method		
Acinetobacter	Acinetobacter	Enterobacter	*Pantoea*
*Alcaligenes*	*Acetobacter*	Enterobacter	*Parabacteroides*
*Bacillus*	*Alcaligenes*	*Enterobacteriaceae*	*Paracoccus*
*Bhargavaea*	*Alkalibacterium*	*Enterococcus*	*Planococcus*
*Brachybacterium*	*Arthrobacter*	Escherichia	*Planomicrobium*
*Chryseobacterium*	*Bacillus*	*Exiguobacterium*	*Porphyromonas*
Enterobacter	*Bacteroides*	*Janibacter*	Pseudomonas
*Enterobacteriaceae*	*Bhargavaea*	Klebsiella	*Serratia*
*Exiguobacterium*	*Bordetella*	*Kocuria*	*Sphingobacterium*
*Janibacter*	*Brachybacterium*	*Kurthia*	Staphylococcus
Klebsiella	*Brachybacterium*	*Kytococcus*	*Stenotrophomonas*
*Micrococcus*	*Brevibacillus*	*Lysinibacillus*	*Streptomyces*
*Myroides*	*Brevundimonas*	*Lysobacter*	*Terribacillus*
*Pantoea*	*Burkholderia*	*Macrococcus*	*Tetrasphaera*
Pseudomonas	*Cellulosimicrobium*	*Micrococcus*	*Thioclava*
Staphylococcus	*Chryseobacterium*	*Myroides*	*Xylella*
*Stenotrophomonas*	*Citrobacter*	*Oceanobacillus*	*Yersinia*
	*Cronobacter*	*Paenibacillus*	
Total=17	Total=53		

**TABLE 4:** Bacterial genera cultivated from leachate using the SD and 2DCS cultivation methods

Genera cultivated by the SD method	Genera cultivated by the 2DCS method		
*Alcaligenes*	*Alcaligenes*	*Exiguobacterium*	*Paracoccus*
*Bacillus*	Acinetobacter	*Geobacillus*	*Planococcus*
*Bacteroides*	*Agrococcus*	*Halobacillus*	*Planomicrobium*
*Brevundimonas*	*Anaplasma*	*Janibacter*	*Porphyromonas*
*Chromobacterium*	*Anoxybacillus*	Klebsiella	*Providencia*
*Citrobacter*	*Bacillus*	*Kocuria*	*Providencia*
*Clostridium*	*Bacteroides*	*Kurthia*	Pseudomonas
*Laribacter*	*Brachybacterium*	*Laribacter*	Pseudomonas
*Lysobacter*	*Brevibacillus*	*Lysinibacillus*	*Renibacterium*
*Parabacteroides*	*Brevibacterium*	*Lysobacter*	*Serratia*
*Porphyromonas*	*Brevundimonas*	*Macrococcus*	*Sphingobacterium*
*Providencia*	*Cellulomonas*	*Methylobacterium*	*Sporosarcina*
Pseudomonas	*Cellulosimicrobium*	*Microbacterium*	Staphylococcus
*Serratia*	*Chromobacterium*	*Micrococcus*	*Stenotrophomonas*
*Sphingobacterium*	*Citrobacter*	*Myroides*	*Streptomyces*
*Thioclava*	*Clavibacter*	*Oceanobacillus*	*Terrabacter*
*Yersinia*	*Clostridium*	*Okibacterium*	*Thioclava*
	*Clostridium*	*Ornithinibacillus*	*Virgibacillus*
	*Desulfuromonas*	*Paenibacillus*	*Xylella*
	Enterobacter	*Pantoea*	*Yersinia*
	*Enterococcus*	*Parabacteroides*	*Zimmermannella*
Total=17	Total=63		

**FIG 3:**
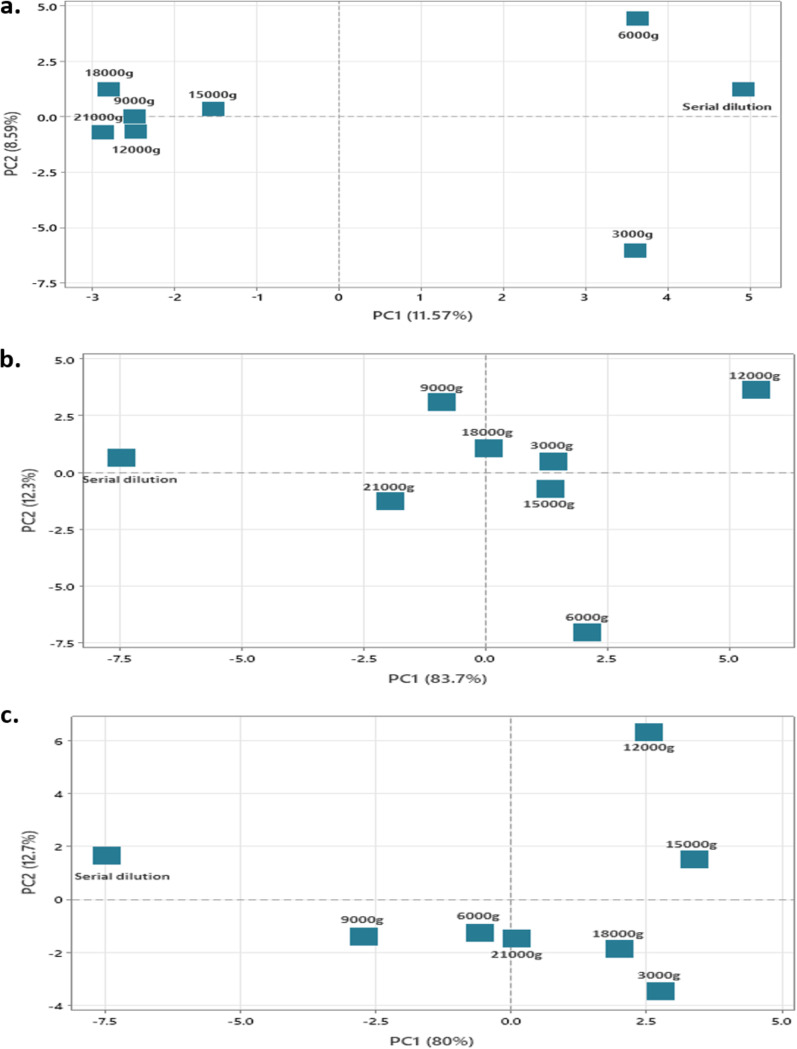
Ordination plot of cultivated microbial community composition at the taxonomic level of family for soil (a), anaerobic sludge (b), and landfill leachate (c). For each data set, a principal-component analysis (PCA) was performed to compare between taxonomic composition recovered using the SD method and at each centrifugal increment for the 2DCS method. Each data point represents all microbial taxa recovered across all dilutions.

The effect of centrifugation force on microbial communities was examined prior to cultivation using microscopy and after cultivation through 16S rRNA gene amplicon sequencing. Cells from the three sample types were clearly observed up to 12,000 × *g*; however, at *g* forces greater than 12,000 × *g*, cells were not observed at ×1,000 magnification, either due to low biomass or the small size of cells remaining in the supernatant. However, cells were recovered and cultivated at the highest centrifugation forces, but total cell numbers were low, and isolates were generally not detected after dilution (e.g., Fig. 2). Differences in the cultivable microbial communities after each centrifugation step were visualized using principal-component analysis (PCA) at the taxonomic level of family) (Fig. 3). In all three sample types (Fig. 3a to c), cultivated microorganisms obtained using the SD method were separated from cultivated microorganisms obtained from different centrifugal force dilutions on PC axis 1 (loading, 11.57% to 83.7%). No consistent trend among the different centrifugal force samples was observed between different sample types.

## DISCUSSION

The ability to isolate representative microorganisms from environmental samples is an important and challenging endeavor. Isolation of microorganisms, coupled with physiological testing and genome assemblies, is critically important to address questions in microbial ecology, assess microbial hazards (e.g., antibiotic resistance), and improve annotation of cultivation-independent metagenomic and metatranscriptomic sequence data (1). However, the gap between known diversity and cultivable microbial diversity has been long known and is well established (9, 10). Various causes of microbial noncultivability include substrate-accelerated death during the transfer of cells from natural, often oligotrophic environments to nutrient-rich synthetic media, lack of adequate or appropriate nutrients, short incubation time favoring fast-growing organisms, disruption of inter- and intraspecies cell signaling and communication, and more (3, 5, 8, 36, 37). Another critical issue with standard cultivation techniques is the competition for nutrients and spaces among fast-growing *r*-strategist organisms and slow-growing *k*-strategist organisms. We have often observed that slow-growing taxa are difficult to isolate using standard agar cultivation due to colony mixing and lawn formation by fast-growing taxa. To address this issue, multiple techniques have been attempted, and prior studies have shown that techniques employing cell separation or isolation followed by growth have generally been more successful in recovery of isolate diversity than cultivation strategies employing mixed growth followed by isolation, including gel encapsulation and cultivation in an I-chip (38, 39). Though these techniques reduce competition and can provide natural nutrients if incubated *in situ* after encapsulation, the application of high temperatures (40°C) at the time of encapsulation of the cells in gelling materials and the demand for custom instrumentation reduce the uptake of these methods.

We sought to develop a novel cultivation method to increase recovery of microbial diversity, but without the need for sophisticated instrumentation. The method demonstrated here employs only standard-cultivation laboratory instrumentation for dilution plating on agar media (i.e., autoclave, biosafety cabinet, incubator) as well as a centrifuge. The method employs sequential centrifugation of microbial cells at increasing *g* forces, followed by serial dilution. As environmental samples contain an array of microbes of different shapes, sizes, and biomass, these cells can be fractionated by sedimentation under different centrifugal forces. Cells will differentially sediment according to biomass, surface area, cell shape, clumping tendency, and buoyancy. Although many cell separation techniques are possible, centrifugation is particularly attractive due to ease of access and simplicity of workflow. We employed incremental steps of 3,000 × *g* up to 21,000 × *g* and were able to recover different microbial taxa at each step. Furthermore, the method proposed here does not expose cells to high temperatures, allowing for recovery of temperature-sensitive cells. Serial dilution after differential centrifugation provides for further separation of cells by abundance.

In our proof-of-concept study, we performed cultivation using the 2DCS method and compared it to standard serial dilution (SD) cultivation from the same samples. We observed that even with the use of only a single medium and cultivation temperature, a roughly 3- to 4-fold increase in unique microbial isolates was obtained with the 2DCS method relative to SD, and this range extended to unique genera and species recoveries as well. This substantial increase in recoverable microbial diversity was observed across all three sample types (soil, leachate, and sludge) and was observed under both aerobic and anaerobic cultivation conditions. Although bacteria from only four phyla were recovered in both 2DCS and SD cultivation efforts, marked increases in the number of relatively rare genera were observed for *Proteobacteria*, *Bacteroidetes*, *Actinobacteria*, and *Firmicutes.* We note that the limited phylum-level diversity is likely a result of the single medium employed (LB agar) and suggest that these results could be extended with the application of multiple media and incubation conditions. Across three high-diversity microbial samples, however, the methodology increased cultivable diversity regardless of sample type or incubation condition. Microbial cultivability can be increased several-fold using the 2DCS approach by using different growth media and incubation conditions, with and without initial heat shock, and more.

During the initial incubation period of 48 h, colonies primarily developed on plates receiving the least diluted samples, while many of the higher-dilution samples did not show any growth. Upon further incubation for 15 days, diverse colony morphotypes were also observed in higher-dilution plates, demonstrating that the gradient centrifugation combined with serial dilution also allows for the growth of slow-growing bacteria due to better separation and reduced competition with fast-growing taxa in traditional serial dilutions. We and others have previously demonstrated the critical importance of long incubation periods for characterization of microbial physiology (5). Here, the fractionation of microorganisms by centrifugation and dilution appears to provide improved opportunities for slow-growing taxa to avoid overgrowth during long incubations. This is a likely significant contributor to the increased microbial diversity recovered using the 2DCS method. We identified a number of representatives of microbial genera that have been poorly recovered elsewhere in a limited proof-of-concept study employing three sample types and a single-cultivation condition. We further note the identification of a number of microbial isolates with 16S rRNA gene sequences that are less than 98.5% similar to described species. The criteria of the novelty of species and genera in the polyphasic approach of bacterial and archaeal taxonomy are <98.5 and <95%, respectively (40), and thus, these isolates may represent novel species. The ratio of recovery of novel taxa was consistently greater for the 2DCS method relative to the SD method. Further manipulation of cultivation conditions and sample types will likely yield a larger number of representatives of these poorly characterized genera for study.

Although the 2DCS method is straightforward to employ and yields significantly higher recovery of microbial diversity, there is some possibility that the high centrifugal forces could modify viability of some microbial taxa. For example, Pembrey et al. and Peterson et al. demonstrated that bacterial cell surface charges, adhesion to solid surfaces (Pseudomonas aeruginosa), virulence (Chlamydia psittaci), and internal structural changes were induced due to the shear forces exerted by high-speed centrifugation (41, 42). The authors also demonstrated a drastic reduction in the viability of Escherichia coli at 15,000 × *g* relative to 5,000 × *g* but no change in the viability of a *Psychrobacter* sp. or of Staphylococcus epidermidis. The production of exopolysaccharide and tough cell walls of Gram-positive bacteria have been suggested to protect them from the damaging effect of shear forces at high centrifugal speed, thus maintaining their viability. Consistent with the findings of Pembrey et al. and Peterson et al., only *Actinobacteria* and exopolysaccharide-producing *Rhizobia* were prevalent at high centrifugal speed (18,000 to 21,000 × *g*) (Tables S4 and S5 in the supplemental material) (41, 42). Selective killing of sensitive taxa by elevated centrifugal forces likely further contributes to the enhanced microbial diversity using the 2DCS method. However, we note that all microbial genera that were recovered using the SD method (without centrifugation) were also recovered using the 2DCS method.

We also demonstrate here that some microbial taxa remain in suspension at centrifugal forces that are commonly used to pellet microbial cells (e.g., 10,000 to 16,000 × *g*). Although cells pelleted at 18,000 and 21,000 × *g* were not visible by microscopy at ×1,000 magnification, the presence of viable cells was demonstrated by inoculation of LB liquid media with cell pellets; this likely reflects the presence of very small cells. Such a finding should be taken into consideration in general microbial studies, including cultivation-independent approaches, where the application of insufficient centrifugal forces during pelleting of cells for nucleic acid extraction may lead to the loss of detectable microbial diversity.

### Conclusion.

The two-dimensional cell separation approach involving segregation of microbial cells based on their sedimentation rate proved to be a novel and efficient technique for high-throughput cultivation of microorganisms. This approach can be incorporated with less labor and complexity in laboratories with minimal instrumentation. As cultivation is a workflow that is well suited to lower-resource microbiology labs, we believe improvements in cultivability can increase opportunities for scientific collaborations between low-resource labs and groups focused on high-resource cultivation-independent methodologies. The microbial diversity captured through this proof-of-concept study with only a single growth medium, and culture condition was 2- to 3-fold higher at the genus level and 3- to 4-fold higher at the species level in comparison to traditional serial dilution to extinction. Cultivability can be enhanced by greater folds with the use of different media and incubation conditions. This increased recovery of microbial taxa was observed across three different sample types and under aerobic and anaerobic cultivation conditions. We also note that microbial cells were retrieved after centrifugation at 18,000 × *g* and 21,000 × *g*; thus, sufficiently high *g* forces should be applied in cell-pelleting approaches coupled to cultivation-independent molecular analyses to ensure maximum representation of community structure.

## MATERIALS AND METHODS

### Environmental sampling.

To assess the cultivation efficiency and reproducibility of the proposed method, three environment samples from different niches (agricultural soil, landfill leachate, and sludge) were selected. Soil was collected from an agricultural field (located in Pimpri-Chinchwad municipal corporation area, Pune, India). Landfill leachate was collected from a landfill site located in Bhandewadi, Nagpur, India. Anaerobic sludge was collected from an optimally operated upflow anaerobic sludge blanket reactor (UASB) from a food processing industry wastewater treatment plant near Nagpur, India. Collected samples were transported to the lab on ice and stored at 4°C. Cultivation experiments from collected samples were started within 4 days of collection to minimize loss of biodiversity.

### Two-dimensional cell separation by differential centrifugation and dilution to extinction.

We suspended 100 mg of soil, leachate, or sludge in 900 μL of sterile normal saline under aseptic conditions. These suspensions were vortexed for 2 min to detach microbial cells from solid particles. Subsequently, each suspension was subjected to sequential rounds of centrifugation at increasing centrifugal forces. For each sample type, 1 mL of the initial suspension was transferred to a fresh tube and centrifuged at 3,000 × *g* for 10 min. The supernatant obtained from each round of centrifugation was subjected to a higher level of centrifugation. For each sample, serial centrifugation was performed from 3,000 × *g* to 21,000 × *g* in increments of 3,000 × *g* (i.e., 3,000 × *g*, 6,000 × *g*, 9,000 × *g*, 12,000 × *g*, 15,000 × *g*, 18,000 × *g*, and 21,000 × *g*). After each transfer of supernatants, the remaining cell pellet was resuspended in 1 mL of normal saline for dilution plating (Fig. 1). Traditional serial dilution (SD) (i.e., dilution to extinction) (43) with the same media and growth conditions but without centrifugation steps was used as a comparator to the novel 2DCS method.

### Cultivation and colony isolation.

One hundred microliters of inoculum from each dilution was plated in triplicate on Luria-Bertani (LB) agar and incubated at 30°C for 24 to 48 h to get visible colony growth. Plates were incubated under aerobic conditions for the soil and leachate samples and anaerobic conditions in an anaerobic jar (Equitron Medica, Mumbai, India) for the anaerobic sludge sample. Colonies with distinct morphotypes were picked and streaked on fresh LB agar plates, purified, and preserved in sterile 1× phosphate-buffered saline (PBS) with 20% glycerol and stored at −20°C (2). Plates were further incubated for 15 days to provide sufficient time for the development of colonies from slow-growing microorganisms, and colonies with distinct morphotypes were processed as described above.

### Microscopy.

Cell pellet suspensions obtained at each step were examined using microscopy and subsequently inoculated in LB broth to confirm cell viability by visible growth. Microscopic observation was carried out to measure the bacterial load remaining after each successive centrifugation. After each transfer, portions of cell pellet suspensions were stained using a Gram-staining protocol and observed with a compound microscope (Olympus BX53 digital microscope) at ×1,000 magnification (10× eyepiece and 100× objective). Microbial cells were not detected in cell pellet suspensions recovered at 12,000 × *g* and higher, either due to low cell abundance and/or ultrasmall size of the cells. Suspensions without visible bacteria were used to inoculate LB broth, with cell growth used to confirm the viability and presence of microbial cells.

### Characterization of microbial isolates from the agricultural soil sample.

For DNA-based sequence identification, colonies were subjected to DNA extraction and PCR with 16S rRNA gene primers (27F, 5′-GAGTTTGATCMTGGCTCAG-3′; 1492R, 5′-TACGGYTACCTTGTTACGA-3′) and sequenced using capillary electrophoresis sequencing on a 3730 DNA analyzer (Thermo Fisher Scientific) as described previously (44). The closest matches to recovered sequences were generated using the BLAST algorithm using the EzBioCloud database (45).

### Bulk characterization of microbial isolates from anaerobic sludge and landfill leachate samples using next-generation sequencing.

The total number of morphotypes obtained from anaerobic sludge and leachate was nearly 1,000, as determined by visual inspection of agar plates. As capillary electrophoresis sequencing of so many isolates is expensive and time-consuming, we sought to design a less expensive and less time-consuming approach. Briefly, each morphologically unique isolate was subcultured in LB broth, and following overnight growth, 10 μL of each culture was pooled in separate sterile Falcon tubes, thus creating 16 composite samples (7 corresponding to isolates from individual steps of the centrifugation step and 1 corresponding to each isolate from the serial dilution method for anaerobic sludge and the leachate samples). Total DNA from 16 pooled cell mixtures was extracted using a FastDNA soil kit (MP Biomedicals, CA, USA) and was quantified using Qubit DNA HS assay. The DNA samples were diluted to a concentration of 5 ng per microliter and were amplified using primers targeting near-full-length 16S rRNA genes (16SF: 5′-AGAGTTTGATCCTGGCTCAG-3′ and 16SR: 5′-GGTTACCTTGTTACGACTT-3′). PCR products were pooled and subjected to nested amplification with domain-level primers targeting the V3-V4 region of 16S rRNA genes (V3V4F, 5′-CCTACGGGNGGCWGCAG-3′, and V3V4R, 5′-GACTACHVGGGTATCTAATCC-3′) followed by clean-up using AMPure XP beads (Beckman Coulter). Library preparation of the cleaned product was performed using NEBNext Ultra DNA library prep kit for Illumina according to the manufacturer’s instructions. Libraries were sequenced on an Illumina MiSeq instrument employing V2 chemistry (2 × 250 paired-end reads). The raw sequencing data were processed to obtain high-quality clean reads using Trimmomatic v0.38 (46) to remove adapter sequences, ambiguous reads, and low-quality sequences. The filtered high-quality reads were assembled into scaffolds using metaSPAdes (47), and obtained sequence data were analyzed using the RDP database on MG-RAST for exploring the culturable bacterial diversity.

### Statistical analysis.

The numbers of isolates and characterized genera and species from all three samples were summed separately. The average and standard deviation of obtained data were calculated in Excel. *P* values and F statistics of the obtained data were calculated using one-way analysis of variance (ANOVA) test. An unweighted principal-component analysis (PCA) was performed using data generated from the pooled 16S rRNA gene amplicon sequence to discriminate the number of family-level taxa obtained from SD and from each centrifugal force used during 2DCS using the software package Minitab (48).

### Data availability.

Raw Illumina sequence data were deposited in the National Center for Biotechnology Information (NCBI) Sequence Read Archive (SRA) under the BioProject identifier PRJNA807079. BioSample and SRA accession numbers for landfill leachate samples are SAMN26003003 to SAMN26003010 and SRX14208010 to SRX14208003, respectively. In addition, BioSample and SRA accession numbers for anaerobic sludge samples are SAMN26002995 to SAMN26003002 and SRX14208012 to SRX14208009, respectively. The 16S rRNA gene sequences of isolates generated using Sanger sequencing were submitted to GenBank with sequence accession numbers from OM971075 to OM971647 (Table S6).
