# Supplementary material for: Two-Dimensional Cell Separation: a High-Throughput Approach to Enhance the Culturability of Bacterial Cells from Environmental Samples
Source: Microbiol Spectr. 2022 Apr 25;10(3):e00007-22. doi: 10.1128/spectrum.00007-22 (PMC9248899; doi:10.1128/spectrum.00007-22)
Supplement: SUPPLEMENTAL FILE 1 — Supplemental material. Download spectrum.00007-22-s001.pdf, PDF file, 0.3 MB [file spectrum.00007-22-s001.pdf]

Supplementary table 1: Bacterial species cultivated from soil using SD and 2DCS cultivation methods.

**Species cultivated by SD**	**Species cultivated by 2DCS**		
Agrobacterium cavarae	*Acinetobacter towneri*	*Lysobacter zhanggongensis*	*Pseudomonas graminis*
Agromyces italicus	*Actinoplanes missouriensis*	*Massilia aerilata*	*Pseudomonas monteilii*
Agromyces laixinhei	*Actinoplanes palleronii*	*Massilia agri*	*Pseudomonas mosselii*
Bacillus cereus	*Actinoplanes subglobosus*	*Massilia albidiflava*	*Pseudomonas neuropathica*
Bacillus coreaensis	*Agrobacterium cavarae*	*Massilia flava*	*Pseudomonas nitrititolerans*
Bacillus pacificus	*Agromyces humi*	*Massilia lutea*	*Pseudomonas plecoglossicida*
Bacillus toyonensis	*Agromyces italicus*	*Massilia oculi*	*Pseudoxanthomonas indica*
Bacillus wiedmannii	*Agromyces laixinhei*	*Massilia timonae*	*Pseudoxanthomonas koreensis*
Brachybacterium paraconglomeratum	*Alcaligenes faecalis subsp. faecalis*	*Massilia umbonata*	*Pseudoxanthomonas mexicana*
Brachybacterium sacelli	*Alkalihalobacillus alkalinitrilicus*	*Mesobacillus boroniphilus*	*Psychrobacillus lasiicapitis*
Brucella cytisi	*Ancylobacter oerskovii*	*Metabacillus halosaccharovorans*	*Psychrobacillus soli*
Cellulomonas denverensis	*Arthrobacter crystallopoietes*	*Metabacillus niabensis*	*Rhizobium alamii*
Cellulomonas pakistanensis	*Arthrobacter globiformis*	*Methylocella tundrae*	*Rhizobium azibense*
Cellulomonas taurus	*Bacillus cereus*	*Microbacterium aerolatum*	*Rhizobium cellulosilyticum*
Cellulosimicrobium funkei	*Bacillus coreaensis*	*Microbacterium aoyamense*	*Rhizobium esperanzae*
Chryseobacterium arthrosphaerae	*Bacillus pacificus*	*Microbacterium arborescens*	*Rhizobium pakistanense*
Chryseobacterium cucumeris	*Bacillus stercoris*	*Microbacterium aurum*	*Rhizobium panacihumi*
Chryseobacterium indologenes	*Bacillus thuringiensis gv. cytolyticus*	*Microbacterium bovistercoris*	*Rhizobium petrolearium*
Erwinia oleae	*Bacillus toyonensis*	*Microbacterium enclense*	*Rhizobium subbaraonis*
Glutamicibacter mishrai	*Bacillus vallismortis*	*Microbacterium*	*Rhizobium wenxiniae*

		esteraromaticum	
Glutamicibacter protophormiae	*Bacillus velezensis*	*Microbacterium foliorum*	*Rhizobium yantingense*
Gordonia terrae	*Bacillus weihaiensis*	*Microbacterium ginsengiterrae*	*Rhizobium zeae*
Isoptericola nanjingensis	*Bacillus wiedmannii*	*Microbacterium gorillae*	*Rhodobacter xinxiangensis*
Luteimonas soli	*Bosea massiliensis*	*Microbacterium hibisci*	*Rhodococcus canchipurensis*
Lysinibacillus fusiformis	*Brachybacterium alimentarium*	*Microbacterium humi*	*Rhodococcus cerastii*
Lysobacter soli	*Brachybacterium endophyticum*	*Microbacterium hydrocarbonoxydans*	*Rhodococcus pedocola*
Lysobacter zhanggongensis	*Brachybacterium paraconglomeratum*	*Microbacterium insulae*	*Rhodococcus rhodochrous*
Metabacillus halosaccharovorans	*Brachybacterium rhamnosum*	*Microbacterium keratanolyticum*	*Shigella flexneri*
Methylocella tundrae	*Brachybacterium sacelli*	*Microbacterium ketosireducens*	*Shinella kummerowiae*
Microbacterium bovistercoris	*Brachybacterium squillarum*	*Microbacterium laevaniformans*	*Shinella zoogloeoides*
Microbacterium esteraromaticum	*Brachymonas chironomi*	*Microbacterium lushaniae*	*Sphingobacterium endophyticum*
Microbacterium foliorum	*Brachymonas denitrificans*	*Microbacterium marinum*	*Sphingobacterium mucilaginosum*
Microbacterium ginsengiterrae	*Brevundimonas olei*	*Microbacterium natoriense*	*Sphingobacterium multivorum*
Microbacterium gorillae	*Brucella cytisi*	*Microbacterium oleivorans*	*Sphingobacterium nematocida*
Microbacterium insulae	*Brucella pseudogrignonensis*	*Microbacterium paraoxydans*	*Sphingobium naphthae*
Microbacterium keratanolyticum	*Cellulomonas denverensis*	*Microbacterium phyllosphaerae*	*Sphingomonas canadensis*
Microbacterium ketosireducens	*Cellulomonas fimi*	*Microbacterium saccharophilum*	*Sphingomonas mucosissima*
Microbacterium laevaniformans	*Cellulomonas pakistanensis*	*Microbacterium sorbitolivorans*	*Sphingomonas yantingensis*
Microbacterium marinum	*Cellulomonas taurus*	*Microbacterium telephonicum*	*Sphingopyxis chilensis*
Microbacterium natoriense	*Cellulosimicrobium cellulans*	*Microbacterium testaceum*	*Sphingopyxis solisilvae*
Microbacterium paraoxydans	*Cellulosimicrobium funkei*	*Microbacterium trichothecenolyticum*	*Sporosarcina luteola*

Neobacillus niacini	*Chryseobacterium arthrosphaerae*	*Microbacterium ureisolvens*	*Stenotrophomonas bentonitica*
Neorhizobium alkalisoli	*Chryseobacterium cucumeris*	*Microbacterium wangchenii*	*Stenotrophomonas chelatiphaga*
Novosphingobium gossypii	*Chryseobacterium flavum*	*Micrococcus luteus*	*Stenotrophomonas indicatrix*
Novosphingobium guangzhouense	*Chryseobacterium indologenes*	*Morganella morganii subsp. sibonii*	*Stenotrophomonas lactitubi*
Novosphingobium resinovorum	*Chryseobacterium timonianum*	*Neobacillus drentensis*	*Stenotrophomonas maltophilia*
Priestia aryabhattai	*Citrobacter braakii*	*Neobacillus niacini*	*Stenotrophomonas nitritireducens*
Priestia filamentosa	*Curtobacterium citreum*	*Neorhizobium alkalisoli*	*Stenotrophomonas panacihumi*
Priestia megaterium	*Cytobacillus firmus*	*Neorhizobium huautlense*	*Stenotrophomonas pavanii*
Pseudochrobactrum asaccharolyticum	*Devosia riboflavina*	*Niallia taxi*	*Stenotrophomonas terrae*
Pseudomonas graminis	*Domibacillus indicus*	*Nitrincola tapanii*	*Streptomyces atriruber*
Pseudomonas monteilii	*Ensifer garamanticus*	*Nocardia carnea*	*Streptomyces atrovirens*
Pseudomonas plecoglossicida	*Ensifer meliloti*	*Nocardia rhamnosiphila*	*Streptomyces badius*
Pseudoxanthomonas indica	*Ensifer terangae*	*Nocardioides lianchengensis*	*Streptomyces bambusae*
Pseudoxanthomonas koreensis	*Enterobacter hormaechei subsp. xiangfangensis*	*Novosphingobium barchaimii*	*Streptomyces bottropensis*
Psychrobacillus lasiicapitis	*Enterobacter quasiroggenkampii*	*Novosphingobium gossypii*	*Streptomyces chrestomyceticus*
Rhizobium panacihumi	*Enterococcus casseliflavus*	*Novosphingobium guangzhouense*	*Streptomyces dioscori*
Rhodococcus cerastii	*Enterococcus durans*	*Novosphingobium resinovorum*	*Streptomyces endophyticus*
Rhodococcus pedocola	*Erwinia oleae*	*Ochrobactrum teleogrylli*	*Streptomyces globisporus*
Rhodococcus rhodochrous	*Glutamicibacter mishrai*	*Paenibacillus pinisoli*	*Streptomyces griseoflavus*
Shigella flexneri	*Glutamicibacter protophormiae*	*Pantoea anthophila*	*Streptomyces griseoviridis*
Sphingobacterium mucilaginosum	*Glutamicibacter uratoxydans*	*Paracoccus lutimaris*	*Streptomyces heliomycini*
Sphingobacterium multivorum	*Gordonia terrae*	*Paucisalibacillus globulus*	*Streptomyces indicus*

Sphingomonas canadensis	*Isoptericola nanjingensis*	*Pedobacter xinjiangensis*	*Streptomyces lavendulae subsp. lavendulae*
Sphingopyxis chilensis	*Janibacter melonis*	*Piscicoccus intestinalis*	*Streptomyces malachitospinus*
Stenotrophomonas indicatrix	*Kineosporia rhizophila*	*Priestia aryabhattai*	*Streptomyces manipurensis*
Stenotrophomonas lactitubi	*Klebsiella quasivariicola*	*Priestia endophytica*	*Streptomyces osmaniensis*
Stenotrophomonas nitritireducens	*Klebsiella variicola subsp. tropica*	*Priestia filamentosa*	*Streptomyces roseifaciens*
Streptomyces chrestomyceticus	*Knoellia locipacati*	*Priestia flexa*	*Streptomyces rubrogriseus*
Streptomyces griseoviridis	*Krasilnikoviella muralis*	*Priestia megaterium*	*Streptomyces scabiei*
Streptomyces malachitospinus	*Leclercia adecarboxylata*	*Pseudarthrobacter enclensis*	*Streptomyces shaanxiensis*
Streptomyces tendae	*Leucobacter musarum subsp. musarum*	*Pseudochrobactrum asaccharolyticum*	*Streptomyces tendae*
Terribacillus saccharophilus	*Leucobacter tardus*	*Pseudoduganella violaceinigra*	*Terribacillus saccharophilus*
	Luteimonas soli	*Pseudomonas asiatica*	*Ureibacillus sinduriensis*
	Lysinibacillus fusiformis	*Pseudomonas entomophila*	*Xanthomonas maliensis*
	Lysobacter soli	*Pseudomonas geniculata*	
**Total = 73**	**Total = 227**		

Supplementary table 2: Bacterial species cultivated from anaerobic sludge using SD and 2DCS cultivation methods.

Species cultivated by SD	Species cultivated by 2DCS		
Acinetobacter calcoaceticus	*Acetobacter pasteurianus*	*Bacillus sp. H-04*	*Micrococcus sp. TS17*
Alcaligenes faecalis	*Acinetobacter johnsonii*	*Bacillus sp. HH-01*	*Oceanobacillus iheyensis*
Bacillus cereus	*Acinetobacter sp.*	*Bacillus sp. KHg1*	*Paenibacillus larvae*
Bacillus cohnii	*Alcaligenes sp.*	*Bacillus sp. KHg2*	*Paenibacillus lentimorbus*
Bacillus firmus	*Alkalibacterium sp. NP13*	*Bacillus sp. NB-6*	*Parabacteroides goldsteinii*
Bacillus licheniformis	*Arthrobacter arilaitensis*	*Bacillus sp. Ob 11*	*Parabacteroides gordonii*
Bacillus pumilus	*Arthrobacter nitroguajacolicus*	*Bacillus sp. PL-12*	*Paracoccus sp. R-24652*
Bacillus sp. Bt 27	*Arthrobacter protophormiae*	*Bacillus sp. SG-1*	*Planococcus sp. L4*
Bacillus sp. SH3	*Arthrobacter sp. FB24*	*Bacillus sp. TT102*	*Planococcus sp. 'SOS Orange'*
Bhargavaea cecembensis	*Bacillus amyloliquefaciens*	*Bacillus sporothermodurans*	*Planomicrobium okeanokoites*
Brachybacterium paraconglomeratum	*Bacillus anthracis*	*Bacillus subtilis*	*Porphyromonas endodontalis*
Chryseobacterium gleum	*Bacillus aquimaris*	*Bacillus thuringiensis*	*Porphyromonas gingivalis*
Enterobacter cloacae	*Bacillus azotoformans*	*bacterium DZY-HS14*	*Pseudomonas pseudoalcaligenes*
Exiguobacterium acetylicum	*Bacillus circulans*	*Bacteroides acidifaciens*	*Pseudomonas sp. S11*
Exiguobacterium aurantiacum	*Bacillus coagulans*	*Bacteroides graminisolvens*	*Pseudomonas xiamenensis*
Exiguobacterium sp. LY3	*Bacillus cytotoxicus*	*Bacteroides thetaiotaomicron*	*Serratia marcescens*
Janibacter sp. BY48	*Bacillus flexus*	*Bordetella sp. IITR02*	*Sphingobacterium multivorum*
Micrococcus luteus	*Bacillus halmapalus*	*Brachybacterium faecium*	*Staphylococcus aureus*
Myroides odoratimimus	*Bacillus halodurans*	*Brevibacillus agri*	*Staphylococcus caprae*
Myroides profundi	*Bacillus horikoshii*	*Brevundimonas bullata*	*Staphylococcus epidermidis*
Pantoea agglomerans	*Bacillus humi*	*Brevundimonas diminuta*	*Staphylococcus hominis*
Pantoea endophytica	*Bacillus infernus*	*Burkholderia cepacia*	*Staphylococcus massiliensis*

Pseudomonas aeruginosa	*Bacillus litoralis*	*Cellulosimicrobium cellulans*	*Staphylococcus schleiferi*
Pseudomonas alcaligenes	*Bacillus macyae*	*Citrobacter freundii*	*Staphylococcus sp. FS-YC6717*
Pseudomonas stutzeri	*Bacillus massiliensis*	*Cronobacter turicensis*	*Staphylococcus warneri*
Staphylococcus arlettae	*Bacillus megaterium*	*Enterobacter amnigenus*	*Staphylococcus xylosus*
Staphylococcus cohnii	*Bacillus methanolicus*	*Enterococcus faecium*	*Streptomyces carpaticus*
Staphylococcus gallinarum	*Bacillus mojavensis*	*Enterococcus malodoratus*	*Streptomyces clavuligerus*
Staphylococcus haemolyticus	*Bacillus mycoides*	*Escherichia coli*	*Streptomyces yeochonensis*
Staphylococcus pasteuri	*Bacillus niacini*	*Escherichia hermannii*	*Terribacillus halophilus*
Staphylococcus saprophyticus	*Bacillus pseudomycoides*	*Kocuria rosea*	*Tetrasphaera veronensis*
Staphylococcus sciuri	*Bacillus psychrosaccharolyticus*	*Kurthia gibsonii*	*Thioclava pacifica*
Stenotrophomonas acidaminiphila	*Bacillus safensis*	*Kurthia sibirica*	*uncultured alpha proteobacterium*
Stenotrophomonas maltophilia	*Bacillus simplex*	*Kurthia zopfii*	*uncultured gamma proteobacterium*
uncultured bacterium	*Bacillus sonorensis*	*Kytococcus sedentarius*	*uncultured proteobacterium*
uncultured Enterobacteriaceae	*Bacillus sp. 3EC2B1*	*Lysinibacillus fusiformis*	*uncultured Pseudomonas sp.*
uncultured Klebsiella sp.	*Bacillus sp. AC-1*	*Lysinibacillus sphaericus*	*uncultured Pseudomonas sp.*
	Bacillus sp. Bt 22	*Lysobacter sp. OC7*	*uncultured Serratia sp.*
	Bacillus sp. BT97	*Lysobacter sp. XL1*	*Xylella fastidiosa*
	Bacillus sp. GB02-14C	*Macrococcus carouselicus*	*Yersinia enterocolitica*
	Bacillus sp. GB02-25	*Micrococcus lylae*	*Yersinia pestis*
	Bacillus sp. GB02-30	*Micrococcus sp. SMCC ZAT351*	
**Total = 37**	**Total = 162**		

Supplementary table 3: Bacterial species cultivated from landfill leachate using SD and 2DCS cultivation methods.

Species cultivated by SD	Species cultivated by 2DCS				
Alcaligenes faecalis	*Alcaligenes faecalis*	*Bacillus niacini*	*Chromobacterium piscinae*	*Micrococcus sp. SMCC ZAT351*	*Staphylococcus cohnii*
Bacillus amyloliquefaciens	*Acinetobacter baumannii*	*Bacillus plakortidis*	*Chryseobacterium gleum*	*Myroides odoratimimus*	*Staphylococcus epidermidis*
Bacillus cereus	*Acinetobacter baylyi*	*Bacillus psychrodurans*	*Chryseobacterium indologenes*	*Myroides profundi*	*Staphylococcus equorum*
Bacillus cohnii	*Acinetobacter calcoaceticus*	*Bacillus psychrosaccharolyticus*	*Citrobacter freundii*	*Oceanobacillus iheyensis*	*Staphylococcus gallinarum*
Bacillus halmapalus	*Acinetobacter haemolyticus*	*Bacillus pumilus*	*Citrobacter gillenii*	*Oceanobacillus picturae*	*Staphylococcus haemolyticus*
Bacillus licheniformis	*Acinetobacter johnsonii*	*Bacillus simplex*	*Citrobacter werkmanii*	*Okibacterium fritillariae*	*Staphylococcus hominis*
Bacillus mycoides	*Acinetobacter sp. 56A1*	*Bacillus smithii*	*Clavibacter michiganensis*	*Ornithinibacillus californiensis*	*Staphylococcus hyicus*
Bacillus sp. GB02-14C	*Agrococcus jenensis*	*Bacillus sonorensis*	*Clostridium botulinum*	*Paenibacillus amylolyticus*	*Staphylococcus massiliensis*
Bacillus sp. HH-01	*Anaplasma phagocytophilum*	*Bacillus sp. 3EC2B1*	*Clostridium drakei*	*Paenibacillus durus*	*Staphylococcus pasteuri*
Bacillus subtilis	*Anoxybacillus flavithermus*	*Bacillus sp. 91*	*Clostridium magnum*	*Paenibacillus illinoisensis*	*Staphylococcus pseudolugdunensis*
Bacillus thuringiensis	*Anoxybacillus kestanbolensis*	*Bacillus sp. Bt 27*	*Clostridium paraputrificum*	*Paenibacillus lentimorbus*	*Staphylococcus saprophyticus*
Bacteroides acidifaciens	*Anoxybacillus pushchinoensis*	*Bacillus sp. BT97*	*Clostridium subterminale*	*Paenibacillus polymyxa*	*Staphylococcus schleiferi*
Brevundimonas bullata	*Bacillus alcalophilus*	*Bacillus sp. GB02-14C*	*Desulfuromonas acetoxidans*	*Paenibacillus sp. KSM-N440*	*Staphylococcus sciuri*
Chromobacterium piscinae	*Bacillus amyloliquefaciens*	*Bacillus sp. GB02-25*	*Enterobacter aerogenes*	*Paenibacillus xylanilyticus*	*Staphylococcus warneri*
Citrobacter freundii	*Bacillus anthracis*	*Bacillus sp. GB02-2A*	*Enterobacter cloacae*	*Paenibacillus zanthoxyli*	*Staphylococcus xylosus*
Citrobacter gillenii	*Bacillus aquimaris*	*Bacillus sp. HH-01*	*Enterobacter hormaechei*	*Pantoea agglomerans*	*Stenotrophomonas acidaminiphila*

Citrobacter werkmanii	*Bacillus azotoformans*	*Bacillus sp. HM06-02*	*Enterococcus faecium*	*Pantoea endophytica*	*Stenotrophomonas maltophilia*
Clostridium subterminale	*Bacillus badius*	*Bacillus sp. JAMB-204*	*Exiguobacterium acetylicum*	*Parabacteroides goldsteinii*	*Streptomyces clavuligerus*
Laribacter hongkongensis	*Bacillus beijingensis*	*Bacillus sp. MB-11*	*Exiguobacterium aurantiacum*	*Parabacteroides gordonii*	*Terrabacter tumescens*
Lysobacter sp. OC7	*Bacillus caldovelox*	*Bacillus sp. MB-5*	*Exiguobacterium sp. AT1b*	*Paracoccus marcusii*	*Thioclava pacifica*
Parabacteroides goldsteinii	*Bacillus catenulatus*	*Bacillus sp. PL-12*	*Exiguobacterium sp. LY3*	*Planococcus sp. PF109*	*uncultured alpha proteobacterium*
Parabacteroides gordonii	*Bacillus cereus*	*Bacillus sp. S210*	*Exiguobacterium undae*	*Planococcus sp. PF8*	*uncultured bacterium*
Porphyromonas endodontalis	*Bacillus circulans*	*Bacillus sp. SH3*	*Geobacillus caldoproteolyticus*	*Planococcus sp. S5*	*uncultured Enterobacteriaceae bacterium*
Providencia rettgeri	*Bacillus clausii*	*Bacillus sp. TT402*	*Halobacillus sp. S19-3*	*Planococcus sp. 'SOS Orange'*	*uncultured gamma proteobacterium*
Pseudomonas sp. 108Z1	*Bacillus cohnii*	*Bacillus sp. w5*	*Janibacter sp. BY48*	*Planomicrobium okeanokoites*	*uncultured Klebsiella sp.*
Pseudomonas xiamenensis	*Bacillus cytotoxicus*	*Bacillus sporothermodurans*	*Klebsiella pneumoniae*	*Porphyromonas endodontalis*	*uncultured marine bacterium*
Serratia marcescens	*Bacillus fastidiosus*	*Bacillus subtilis*	*Kocuria rhizophila*	*Providencia rettgeri*	*uncultured organism*
Serratia proteamaculans	*Bacillus firmus*	*Bacillus thuringiensis*	*Kurthia gibsonii*	*Pseudomonas aeruginosa*	*uncultured proteobacterium*
Sphingobacterium multivorum	*Bacillus flexus*	*Bacillus vietnamensis*	*Laribacter hongkongensis*	*Pseudomonas plecoglossicida*	*uncultured Pseudomonas sp.*
Thioclava pacifica	*Bacillus ginsengi*	*Bacteroides acidifaciens*	*Lysinibacillus fusiformis*	*Pseudomonas putida*	*unidentified proteobacterium*
uncultured alpha proteobacterium	*Bacillus halmapalus*	*Brachybacterium paraconglomeratum*	*Lysinibacillus sphaericus*	*Pseudomonas sp. 108Z1*	*Virgibacillus halodenitrificans*
uncultured bacterium	*Bacillus halodurans*	*Brevibacillus agri*	*Lysobacter sp. OC7*	*Pseudomonas stutzeri*	*Virgibacillus halophilus*
uncultured organism	*Bacillus humi*	*Brevibacillus brevis*	*Macrococcus carouselicus*	*Pseudomonas xiamenensis*	*Virgibacillus koreensis*

Yersinia enterocolitica	*Bacillus hwajinpoensis*	*Brevibacterium casei*	*Methylobacterium rhodinum*	*Renibacterium salmoninarum*	*Virgibacillus marismortui*
Yersinia massiliensis	*Bacillus lentus*	*Brevibacterium linens*	*Microbacterium aurum*	*Serratia marcescens*	*Virgibacillus pantothenticus*
Yersinia pseudotuberculosis	*Bacillus licheniformis*	*Brevibacterium sp. II*	*Microbacterium dextranolyticum*	*Serratia proteamaculans*	*Virgibacillus proomii*
	Bacillus litoralis	*Brevundimonas bullata*	*Microbacterium oxydans*	*Sphingobacterium multivorum*	*Xylella fastidiosa*
	Bacillus marisflavi	*Cellulomonas bogoriensis*	*Microbacterium phyllosphaerae*	*Sporosarcina ureae*	*Yersinia enterocolitica*
	Bacillus megaterium	*Cellulomonas uda*	*Microbacterium sp. D1-15*	*Staphylococcus arlettae*	*Yersinia massiliensis*
	Bacillus mojavensis	*Cellulosimicrobium cellulans*	*Micrococcus luteus*	*Staphylococcus aureus*	*Yersinia pseudotuberculosis*
	Bacillus mycoides	*Cellulosimicrobium sp. HY-13*	*Micrococcus lylae*	*Staphylococcus chromogenes*	*Zimmermannella faecalis*
**Total = 36**	**Total = 205**				

Supplementary table 4: Different type of bacterial genera isolated from agricultural soil sample with application of increasing centrifugal 'g' force

Serial dilution / 0g	3000g	6000g	9000g	12000g	15000g	18000g	21000g
Agrobacterium	*Acinetobacter*	*Actinoplanes*	*Actinoplanes*	*Actinoplanes*	*Actinoplanes*	*Alcaligenes*	*Agromyces*
Agromyces	*Agromyces*	*Agromyces*	*Arthrobacter*	*Ancylobacter*	*Alcaligenes*	*Arthrobacter*	*Arthrobacter*
Bacillus	*Arthrobacter*	*Alkalihalobacillus*	*Bacillus*	*Arthrobacter*	*Arthrobacter*	*Bosea*	*Cellulosimicrobium*
Brachybacterium	*Bacillus*	*Bacillus*	*Brachybacterium*	*Bacillus*	*Brachybacterium*	*Domibacillus*	*Citrobacter*
Brucella	*Brachybacterium*	*Cellulomonas*	*Cellulomonas*	*Brachybacterium*	*Cellulomonas*	*Glutamicibacter*	*Microbacterium*
Cellulomonas	*Brachymonas*	*Cellulosimicrobium*	*Devosia*	*Glutamicibacter*	*Cellulosimicrobium*	*Lysobacter*	*Neobacillus*
Cellulosimicrobium	*Brevundimonas*	*Chryseobacterium*	*Ensifer*	*Isoptericola*	*Enterobacter*	*Massilia*	*Neorhizobium*
Chryseobacterium	*Brucella*	*Cytobacillus*	*Enterococcus*	*Kineosporia*	*Enterococcus*	*Mesobacillus*	*Novosphingobium*
Erwinia	*Cellulosimicrobium*	*Ensifer*	*Janibacter*	*Klebsiella*	*Gordonia*	*Microbacterium*	*Piscicoccus*
Glutamicibacter	*Chryseobacterium*	*Enterobacter*	*Klebsiella*	*Lysobacter*	*Lysobacter*	*Neorhizobium*	*Rhodococcus*
Gordonia	*Curtobacterium*	*Glutamicibacter*	*Krasilnikoviella*	*Metabacillus*	*Massilia*	*Novosphingobium*	*Streptomyces*
Isoptericola	*Knoellia*	*Isoptericola*	*Massilia*	*Microbacterium*	*Microbacterium*	*Pseudomonas*	
Luteimonas	*Leucobacter*	*Leclercia*	*Microbacterium*	*Neorhizobium*	*Neorhizobium*	*Pseudoxanthomonas*	
Lysinibacillus	*Lysinibacillus*	*Lysobacter*	*Micrococcus*	*Pseudoxanthomonas*	*Novosphingobium*	*Rhizobium*	
Lysobacter	*Lysobacter*	*Massilia*	*Neorhizobium*	*Rhizobium*	*Pseudomonas*	*Shinella*	
Metabacillus	*Microbacterium*	*Microbacterium*	*Novosphingobium*	*Rhodobacter*	*Psychrobacillus*	*Sphingobacterium*	
Methylocella	*Morganella*	*Micrococcus*	*Paracoccus*	*Sphingomonas*	*Rhizobium*	*Stenotrophomonas*	
Microbacterium	*Niallia*	*Neobacillus*	*Paucisalibacillus*	*Stenotrophomonas*	*Rhodococcus*	*Ureibacillus*	
Neobacillus	*Nocardia*	*Neorhizobium*	*Pedobacter*	*Streptomyces*	*Sphingobacterium*		
Neorhizobium	*Ochrobactrum*	*Nitrincola*	*Pseudoduganella*	*Xanthomonas*	*Streptomyces*		

Novosphingobium	*Paenibacillus*	*Nocardioides*	*Pseudoxanthomonas*		*Xanthomonas*		
Priestia	*Priestia*	*Novosphingobium*	*Rhizobium*				
Pseudochrobactrum	*Pseudomonas*	*Pantoea*	*Sphingobacterium*				
Pseudomonas	*Pseudoxanthomonas*	*Priestia*	*Streptomyces*				
Pseudoxanthomonas	*Psychrobacillus*	*Pseudarthrobacter*					
Psychrobacillus	*Rhizobium*	*Pseudomonas*					
Rhizobium	*Sphingobacterium*	*Pseudoxanthomonas*					
Rhodococcus	*Sphingobium*	*Psychrobacillus*					
Shigella	*Sphingopyxis*	*Rhizobium*					
Sphingobacterium	*Stenotrophomonas*	*Rhodococcus*					
Sphingomonas	*Streptomyces*	*Sphingobacterium*					
Sphingopyxis		*Sphingopyxis*					
Stenotrophomonas		*Sporosarcina*					
Streptomyces		*Stenotrophomonas*					
Terribacillus		*Streptomyces*					
		Terribacillus					
**Total = 35**	**Total = 31**	**Total = 36**	**Total = 24**	**Total = 20**	**Total = 21**	**Total = 18**	**Total = 11**

Supplementary table 5: Different type of bacterial species isolated from agricultural soil sample with application of increasing centrifugal 'g' force

Control/0g	3000g	6000g	9000g	12000g	15000g	18000g	21000g
Agrobacterium cavarae	*Acinetobacter towneri*	*Actinoplanes subglobosus*	*Actinoplanes missouriensis*	*Actinoplanes missouriensis*	*Actinoplanes palleronii*	*Alcaligenes faecalis subsp. faecalis*	*Agromyces humi*
Agromyces italicus	*Agromyces italicus*	*Agromyces italicus*	*Actinoplanes subglobosus*	*Ancylobacter oerskovii*	*Alcaligenes faecalis subsp. faecalis*	*Arthrobacter crystallopoietes*	*Arthrobacter crystallopoietes*
Agromyces laixinhei	*Arthrobacter globiformis*	*Alkalihalobacillus alkalinitrilicus*	*Arthrobacter globiformis*	*Arthrobacter crystallopoietes*	*Arthrobacter crystallopoietes*	*Bosea massiliensis*	*Cellulosimicrobium cellulans*
Bacillus cereus	*Bacillus stercoris*	*Bacillus cereus*	*Bacillus toyonensis*	*Bacillus toyonensis*	*Brachybacterium sacelli*	*Domibacillus indicus*	*Citrobacter braakii*
Bacillus coreaensis	*Bacillus thuringiensis gv. cytolyticus*	*Bacillus pacificus*	*Brachybacterium alimentarium*	*Brachybacterium rhamnosum*	*Cellulomonas fimi*	*Glutamicibacter protophormiae*	*Microbacterium marinum*
Bacillus pacificus	*Bacillus toyonensis*	*Bacillus stercoris*	*Brachybacterium endophyticum*	*Brachybacterium sacelli*	*Cellulosimicrobium funkei*	*Glutamicibacter uratoxydans*	*Microbacterium paraoxydans*
Bacillus toyonensis	*Bacillus velezensis*	*Bacillus toyonensis*	*Brachybacterium rhamnosum*	*Glutamicibacter uratoxydans*	*Enterobacter quasiroggenkampii*	*Lysobacter soli*	*Microbacterium trichothecenolyticum*
Bacillus wiedmannii	*Bacillus weihaiensis*	*Bacillus vallismortis*	*Brachybacterium sacelli*	*Isoptericola nanjingensis*	*Enterococcus durans*	*Massilia aerilata*	*Neobacillus niacini*
Brachybacterium paraconglomeratum	*Bacillus wiedmannii*	*Bacillus wiedmannii*	*Brachybacterium squillarum*	*Kineosporia rhizophila*	*Gordonia terrae*	*Mesobacillus boroniphilus*	*Neorhizobium alkalisoli*
Brachybacterium sacelli	*Brachybacterium endophyticum*	*Cellulomonas pakistanensis*	*Cellulomonas fimi*	*Klebsiella variicola subsp. tropica*	*Lysobacter soli*	*Microbacterium aoyamense*	*Novosphingobium barchaimii*
Brucella cytisi	*Brachybacterium paraconglomeratum*	*Cellulosimicrobium cellulans*	*Devosia riboflavina*	*Lysobacter soli*	*Massilia umbonata*	*Microbacterium foliorum*	*Piscicoccus intestinalis*
Cellulomonas denverensis	*Brachybacterium sacelli*	*Cellulosimicrobium funkei*	*Ensifer garamanticus*	*Metabacillus niabensis*	*Microbacterium aerolatum*	*Microbacterium hibisci*	*Rhodococcus pedocola*
Cellulomonas pakistanensis	*Brachymonas chironomi*	*Chryseobacterium timonianum*	*Enterococcus casseliflavus*	*Microbacterium bovistercoris*	*Microbacterium aurum*	*Microbacterium lushaniae*	*Streptomyces atrovirens*
Cellulomonas taurus	*Brachymonas denitrificans*	*Cytobacillus firmus*	*Janibacter melonis*	*Microbacterium enclense*	*Microbacterium foliorum*	*Neorhizobium alkalisoli*	*Streptomyces osmaniensis*

Cellulosimicrobium funkei	*Brevundimonas olei*	*Ensifer meliloti*	*Klebsiella quasivariicola*	*Microbacterium esteraromaticum*	*Microbacterium humi*	*Novosphingobium barchaimii*	*Streptomyces tendae*
Chryseobacterium arthrosphaerae	*Brucella pseudogrignonensis*	*Ensifer terangae*	*Krasilnikoviella muralis*	*Microbacterium lushaniae*	*Microbacterium oleivorans*	*Novosphingobium gossypii*	
Chryseobacterium cucumeris	*Cellulosimicrobium cellulans*	*Enterobacter hormaechei subsp. xiangfangensis*	*Massilia agri*	*Microbacterium natoriense*	*Microbacterium trichothecenolyticum*	*Pseudomonas entomophila*	
Chryseobacterium indologenes	*Chryseobacterium arthrosphaerae*	*Glutamicibacter protophormiae*	*Massilia oculi*	*Microbacterium oleivorans*	*Microbacterium wangchenii*	*Pseudomonas geniculata*	
Erwinia oleae	*Chryseobacterium cucumeris*	*Glutamicibacter uratoxydans*	*Massilia timonae*	*Microbacterium paraoxydans*	*Neorhizobium alkalisoli*	*Pseudoxanthomonas indica*	
Glutamicibacter mishrai	*Chryseobacterium flavum*	*Isoptericola nanjingensis*	*Microbacterium aurum*	*Microbacterium saccharophilum*	*Neorhizobium huautlense*	*Rhizobium cellulosilyticum*	
Glutamicibacter protophormiae	*Curtobacterium citreum*	*Leclercia adecarboxylata*	*Microbacterium bovistercoris*	*Microbacterium telephonicum*	*Novosphingobium resinovorum*	*Rhizobium pakistanense*	
Gordonia terrae	*Knoellia locipacati*	*Lysobacter soli*	*Microbacterium hydrocarbonoxydans*	*Microbacterium wangchenii*	*Pseudomonas entomophila*	*Rhizobium subbaraonis*	
Isoptericola nanjingensis	*Leucobacter musarum subsp. musarum*	*Massilia albidiflava*	*Microbacterium marinum*	*Neorhizobium alkalisoli*	*Pseudomonas plecoglossicida*	*Rhizobium wenxiniae*	
Luteimonas soli	*Leucobacter tardus*	*Massilia flava*	*Microbacterium natoriense*	*Pseudoxanthomonas mexicana*	*Psychrobacillus soli*	*Rhizobium yantingense*	
Lysinibacillus fusiformis	*Lysinibacillus fusiformis*	*Massilia lutea*	*Microbacterium oleivorans*	*Rhizobium alamii*	*Rhizobium pakistanense*	*Shinella kummerowiae*	
Lysobacter soli	*Lysobacter soli*	*Microbacterium arborescens*	*Microbacterium testaceum*	*Rhizobium azibense*	*Rhizobium panacihumi*	*Shinella zoogloeoides*	
Lysobacter zhanggongensis	*Microbacterium foliorum*	*Microbacterium enclense*	*Micrococcus luteus*	*Rhizobium esperanzae*	*Rhizobium zeae*	*Sphingobacterium mucilaginosum*	
Metabacillus halosaccharovorans	*Microbacterium keratanolyticum*	*Microbacterium esteraromaticum*	*Neorhizobium alkalisoli*	*Rhizobium pakistanense*	*Rhodococcus canchipurensis*	*Stenotrophomonas pavanii*	
Methylocella tundrae	*Microbacterium ketosireducens*	*Microbacterium foliorum*	*Neorhizobium huautlense*	*Rhizobium panacihumi*	*Sphingobacterium mucilaginosum*	*Streptomyces globisporus*	

Microbacterium bovistercoris	*Microbacterium laevaniformans*	*Microbacterium hibisci*	*Novosphingobium gossypii*	*Rhizobium wenxiniae*	*Streptomyces dioscori*	*Ureibacillus sinduriensis*	
Microbacterium esteraromaticum	*Microbacterium paraoxydans*	*Microbacterium hydrocarbonoxydans*	*Paracoccus lutimaris*	*Rhizobium yantingense*	*Streptomyces roseifaciens*		
Microbacterium foliorum	*Microbacterium sorbitolivorans*	*Microbacterium keratanolyticum*	*Paucisalibacillus globulus*	*Rhodobacter xinxiangensis*	*Streptomyces tendae*		
Microbacterium ginsengiterrae	*Microbacterium testaceum*	*Microbacterium ketosireducens*	*Pedobacter xinjiangensis*	*Sphingomonas mucosissima*	*Xanthomonas maliensis*		
Microbacterium gorillae	*Microbacterium ureisolvens*	*Microbacterium paraoxydans*	*Pseudoduganella violaceinigra*	*Sphingomonas yantingensis*			
Microbacterium insulae	*Morganella morganii subsp. sibonii*	*Microbacterium phyllosphaerae*	*Pseudoxanthomonas koreensis*	*Stenotrophomonas bentonitica*			
Microbacterium keratanolyticum	*Niallia taxi*	*Microbacterium trichothecenolyticum*	*Rhizobium pakistanense*	*Streptomyces atrovirens*			
Microbacterium ketosireducens	*Nocardia carnea*	*Micrococcus luteus*	*Rhizobium panacihumi*	*Streptomyces dioscori*			
Microbacterium laevaniformans	*Nocardia rhamnosiphila*	*Neobacillus drentensis*	*Rhizobium petrolearium*	*Streptomyces griseoflavus*			
Microbacterium marinum	*Ochrobactrum teleogrylli*	*Neorhizobium alkalisoli*	*Rhizobium subbaraonis*	*Streptomyces griseoviridis*			
Microbacterium natoriense	*Paenibacillus pinisoli*	*Nitrincola tapanii*	*Rhizobium yantingense*	*Streptomyces heliomycini*			
Microbacterium paraoxydans	*Priestia aryabhattai*	*Nocardioides lianchengensis*	*Sphingobacterium mucilaginosum*	*Streptomyces manipurensis*			
Neobacillus niacini	*Priestia filamentosa*	*Novosphingobium barchaimii*	*Stenotrophomonas panacihumi*	*Streptomyces scabiei*			
Neorhizobium alkalisoli	*Priestia megaterium*	*Novosphingobium resinovorum*	*Streptomyces atrovirens*	*Xanthomonas maliensis*			
Novosphingobium gossypii	*Pseudomonas asiatica*	*Pantoea anthophila*	*Streptomyces bambusae*				
Novosphingobium guangzhouense	*Pseudomonas entomophila*	*Priestia aryabhattai*	*Streptomyces bottropensis*				

Novosphingobium resinovorum	*Pseudomonas monteilii*	*Priestia endophytica*	*Streptomyces heliomycini*				
Priestia aryabhattai	*Pseudomonas mosselii*	*Priestia filamentosa*	*Streptomyces tendae*				
Priestia filamentosa	*Pseudomonas nitrititolerans*	*Priestia flexa*					
Priestia megaterium	*Pseudoxanthomonas indica*	*Priestia megaterium*					
Pseudochrobactrum asaccharolyticum	*Psychrobacillus lasiicapitis*	*Pseudarthrobacter enclensis*					
Pseudomonas graminis	*Rhizobium cellulosilyticum*	*Pseudomonas asiatica*					
Pseudomonas monteilii	*Rhizobium panacihumi*	*Pseudomonas geniculata*					
Pseudomonas plecoglossicida	*Rhizobium subbaraonis*	*Pseudomonas neuropathica*					
Pseudoxanthomonas indica	*Sphingobacterium mucilaginosum*	*Pseudoxanthomonas indica*					
Pseudoxanthomonas koreensis	*Sphingobacterium multivorum*	*Psychrobacillus lasiicapitis*					
Psychrobacillus lasiicapitis	*Sphingobacterium nematocida*	*Rhizobium cellulosilyticum*					
Rhizobium panacihumi	*Sphingobium naphthae*	*Rhizobium pakistanense*					
Rhodococcus cerastii	*Sphingopyxis chilensis*	*Rhizobium panacihumi*					
Rhodococcus pedocola	*Stenotrophomonas bentonitica*	*Rhizobium subbaraonis*					
Rhodococcus rhodochrous	*Stenotrophomonas chelatiphaga*	*Rhizobium wenxiniae*					
Shigella flexneri	*Streptomyces atriruber*	*Rhizobium yantingense*					

Sphingobacterium mucilaginosum	*Streptomyces atrovirens*	*Rhizobium zeae*					
Sphingobacterium multivorum	*Streptomyces badius*	*Rhodococcus cerastii*					
Sphingomonas canadensis	*Streptomyces chrestomyceticus*	*Rhodococcus pedocola*					
Sphingopyxis chilensis	*Streptomyces endophyticus*	*Sphingobacterium endophyticum*					
Stenotrophomonas indicatrix	*Streptomyces griseoviridis*	*Sphingobacterium mucilaginosum*					
Stenotrophomonas lactitubi	*Streptomyces lavendulae subsp. lavendulae*	*Sphingobacterium multivorum*					
Stenotrophomonas nitritireducens	*Streptomyces malachitospinus*	*Sphingopyxis solisilvae*					
Streptomyces chrestomyceticus	*Streptomyces rubrogriseus*	*Sporosarcina luteola*					
Streptomyces griseoviridis	*Streptomyces tendae*	*Stenotrophomonas maltophilia*					
Streptomyces malachitospinus		*Stenotrophomonas panacihumi*					
Streptomyces tendae		*Stenotrophomonas terrae*					
Terribacillus saccharophilus		*Streptomyces atrovirens*					
		Streptomyces chrestomyceticus					
		Streptomyces heliomycini					
		Streptomyces indicus					
		Streptomyces shaanxiensis					

		Terribacillus saccharophilus					
**Total = 73**	**Total = 70**	**Total = 78**	**Total = 47**	**Total = 43**	**Total = 33**	**Total = 30**	**Total = 15**
